# Maternal antioxidant treatment partially rescues developmental defects in a *Drosophila* Polr1D mutant model

**DOI:** 10.17912/micropub.biology.001634

**Published:** 2025-06-12

**Authors:** Bridget M. Walker, Caryn N. Zimmerman, Katherine M. Caruth, Bruce A. Knutson, Ryan J. Palumbo

**Affiliations:** 1 Department of Biochemistry & Molecular Biology, SUNY Upstate Medical University, Syracuse, New York, United States

## Abstract

*Drosophila melanogaster* is a versatile *in vivo* platform for small-molecule screening across many disease models. Here, we utilized a *Drosophila* model carrying a clinically relevant mutation in Polr1D to test the antioxidant N-acetyl- *L* -cysteine (NAC) for therapeutic potential. Treating heterozygous *Polr1D* mothers with NAC partially suppressed the developmental defects of their homozygous *Polr1D* mutant larvae. These findings are consistent with antioxidant rescue effects observed in zebrafish and mouse models of Treacher Collins Syndrome (TCS). Our results demonstrate the value of a *Polr1D* mutant *Drosophila* model for identifying chemical suppressors and accelerating the discovery of promising therapeutics in disorders like TCS.

**Figure 1. Developmental arrest of Polr1D mutant larvae is partially rescued by maternal NAC treatment.**
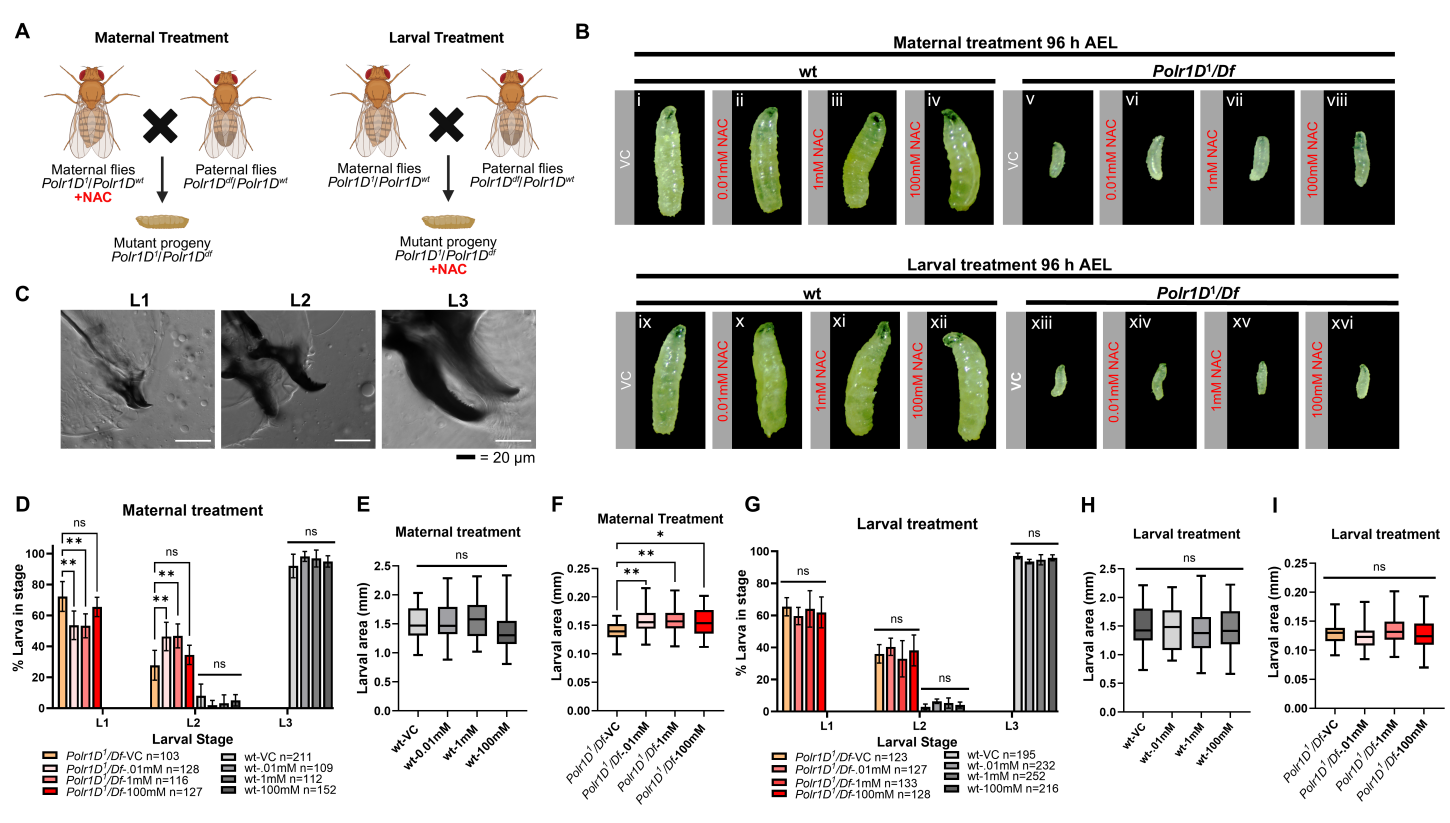
**(A)** Schematic representing the maternal treatment protocol (left), where female flies are given NAC-supplemented food immediately after eclosion, and received fresh NAC every 24 hours prior to crossing to males. Larval treatment protocol (right), where larvae are treated with NAC at 36 h AEL until 96 h AEL. The developmental stage of larvae progeny was determined by mouth hook analysis and measuring larval size. **(B)** Representative images of maternally treated wild-type (wt) (i-iv) and maternally treated *Polr1D ^1^ /+* progeny *Polr1D ^1^ /Df* larvae (v-viii). Images of the larvally treated wt (ix-xii) and *Polr1D ^1^ /Df* progeny (xiii-xvi) at 96 h AEL. Wt larvae are in the L3 stage as expected, while *Polr1D ^1^ /Df* larvae are stalled in the L1 or L2 stage. **(C)** Representative images of cuticles from wt larvae at 96 h AEL representing L1, L2, L3 mouth hooks, used for determining the stage by mouth hook analysis. **(D)** Quantification of the stage of wt and *Polr1D ^1^ /Df* larval progeny of mothers treated with NAC as determined by mouth hook analysis. Treatment of *Polr1D ^1^ /+* mothers with 0.01 mM and 1mM NAC increases the percentage of *Polr1D ^1^ /Df* larvae that progress from the L1 to the L2 stage as compared to treatment with vehicle control. Quantification of the area of **(E)** wt **(F)** *Polr1D ^1^ /Df* larval progeny of mothers treated with NAC. All concentrations of NAC significantly increase *Polr1D ^1^ /Df* larval area as compared to treatment with vehicle control. **(G)** Quantification of the stage of wt and *Polr1D ^1^ /Df* larval progeny of larvally treated with NAC as determined by mouth hook analysis. None of the *Polr1D ^1^ /Df* NAC treated larvae increase the progression from the L1 to the L2 stage. Quantification of the area of larvally treated **(H)** wt and **(I)** *Polr1D ^1^ /Df* progeny. None of the *Polr1D ^1^ /Df* NAC treated larvae increase in size. Statistical significance in D-I was calculated using Prism (GraphPad, version 10.0) by two-way ANOVA followed by Dunnett's multiple comparisons test. A single pooled variance was used for all comparisons. *P* values of <.05 (*), <0.01 (**) and not significant (ns) are indicated.

## Description

*Drosophila melanogaster* has emerged as a powerful *in vivo* platform for small-molecule suppressor screens across many disease models. In the *Drosophila* model of Parkinson's disease caused by mutations in Vesicular monoamine transporter (Vmat), a chemical library screen identified novel psychotropic and neuroprotective drugs (Lawal et al., 2014). Additionally, in the *Drosophila* model of Huntington's disease caused by expanded poly-Q tracts in the Huntingtin protein, a screen using established pharmacological agents identified several compounds that increase longevity (Schulte et al., 2011). Finally, a *Drosophila* Ras-driven cancer model has been utilized to uncover effective combinatorial drug treatment strategies (La Marca et al., 2023). *Drosophila* models have clear phenotypic readouts and scalability which allows them to fill the critical niche between cell-based assays and mammalian studies.

Previously, we established *Drosophila* as a model to study mutations in the RNA polymerase (Pol) I and III subunit POLR1D (Palumbo et al., 2022). POLR1D, along with its binding partner POLR1C, are essential for Pol I and III transcription of rRNAs, and subsequent ribosome biogenesis (Laferte et al., 2006; Wild & Cramer, 2012). Mutations in *POLR1D* and *POLR1C* , as well as *TCOF1* (which encodes Treacle, a Pol I transcription factor), cause Treacher Collins Syndrome (TCS; Dauwerse et al., 2011; Trainor et al., 2009). TCS is a craniofacial disorder caused by reduced neural crest cell (NCC) viability during embryonic development (Trainor, 2010). We sequenced the *Polr1D ^1^* allele in a publicly available fly line and identified a single point mutation that causes a glycine to arginine (G30R) substitution in the highly conserved alpha motif of Polr1D (Palumbo et al., 2022). The G30R substitution occurs at a highly conserved glycine that is mutated in a TCS patient to glutamate (G52E; Vincent et al., 2016). In *Drosophila* this mutation causes larval developmental arrest and growth defects, making the *Polr1D* mutant allele a highly relevant model for studying the developmental defects caused by mutations in clinically relevant residues of a protein mutated in TCS (Palumbo et al., 2022; Walker et al., 2025).

The developmental defects in TCS have been linked to elevated levels of reactive oxygen species (ROS), which play a significant role in the cellular stress experienced by NCCs during their differentiation from the neuroepithelium (Sakai et al., 2016). In both mouse *TCOF1* haploinsufficiency and zebrafish *polr1c* knockdown models of TCS, NCCs undergo apoptosis due to the failure to repair ROS-induced DNA damage (Sakai et al., 2016; Ulhaq et al., 2024). Treacle, in addition to promoting Pol I activity, also protects DNA from ROS through interactions with the DNA damage sensing complex MRNM (Sakai et al., 2016). POLR1D and POLR1C help to mitigate ROS damage indirectly by maintaining nucleolar structure and the translation of DNA damage response proteins (Dash et al., 2023; Ni & Buszczak, 2023; Sakthivel et al., 2023). Interestingly, apoptosis in these models was suppressed by treatment with the antioxidant N-acetyl- *L* -cysteine (NAC), suggesting that ROS contributes to the developmental defects observed in TCS and highlighting antioxidants as a potential therapeutic approach to promote normal development (Sakai et al., 2016; Ulhaq et al., 2024).

While zebrafish and mouse models are currently the most widely used to study the developmental defects underlying TCS, they both require considerable investment of resources and time. Additionally, these models make use of complete deletions or knockdowns of *TCOF1* , *POLR1D* , and *POLR1C* , which prevent the evaluation of chemical suppressors of phenotypes caused by specific TCS point mutations (Dixon et al., 2006; Kwong et al., 2018; Noack Watt et al., 2016). Here, we determined the efficacy of NAC in a *Polr1D* mutant model and provide two treatment protocols to utilize *Drosophila* as a TCS chemical suppressor screening tool.

In mouse models, *in utero* injections of wild type (wt) mothers with NAC improved the craniofacial development of their *TCOF1 ^+/-^* pups (Sakai et al., 2016). *Drosophila Polr1D* mutants are arrested as late as the L2 larval stage and exhibit growth defects (Palumbo et al., 2022; Walker et al., 2025). Therefore, we asked whether treatment of heterozygous *Polr1D ^1^ /+* mothers with NAC rescues the developmental arrest and growth defects of their *Polr1D* mutant (“ *Polr1D ^1^ /Df* ”) progeny. To test this, we raised wt and *Polr1D ^1^ /+* females on food containing multiple concentrations of NAC or vehicle control (1X PBS) ( **Figure 1A left** ) and observed the development and growth of their wt and *Polr1D* mutant progeny, respectively, until 96 h after egg-laying (AEL; **Figure 1B i-viii** ). To determine larval stage, we analyzed larval mouth hooks, which exhibit distinct morphologies at each larval stage ( **Figure 1C** ) (Bodenstein, 1950).

Treating *Polr1D ^1^ /+* mothers with 0.01 mM and 1 mM NAC yielded significantly more *Polr1D* mutant progeny that molted from the L1 larval stage to the L2 larval stage ( **Figure 1D** ). Treatment with 0.01 mM NAC led to a 1.67-fold increase in L2 larvae ( *p* = .0026), and treatment with 1 mM NAC led to a 1.68-fold increase in L2 larvae ( *p* = .0022). Interestingly, treatment with 100 mM NAC did not lead to a significant increase in L2 larvae ( *p* = .2375), yet treating wt mothers with NAC at any concentration did not affect the development of their wt progeny ( **Figure 1B i-iv, D, E** ). These observations suggest the possibility that larval development of *Polr1D* mutants might be particularly sensitive to treatment of *Polr1D ^1^ /+* mothers with especially high concentrations of NAC (Shaposhnikov et al., 2018). Nevertheless, treating *Polr1D ^1^ /+* mothers with all three concentrations of NAC lead to a significant increase in the size of their *Polr1D* mutant larval progeny (100 mM, *p* = .0286; 1 mM, *p =* .0069; 0.01 mM, *p* = .0052; **Figure 1F** ). Interestingly, maternal NAC treatment did not promote development of *Polr1D* mutant larvae beyond the L2 stage, suggesting that there are other factors at play that affect later stages of larval development. Nevertheless, maternal antioxidant treatment partially rescues *Polr1D* mutant larval developmental arrest and growth defects, analogous to the rescue observed in the *TCOF1* haploinsufficiency model (Sakai et al., 2016). Our findings further support the idea that maternal treatment with antioxidants protects embryos from the fatal effects of ROS during embryogenesis.

In zebrafish, knockdown of *polr1c* with morpholino oligonucleotides causes craniofacial defects, recapitulating what is observed in humans. Treating *polr1c* knockdown zebrafish embryos and early larvae with NAC suppressed the craniofacial defects (Ulhaq et al., 2024). Therefore, we investigated whether NAC treatment could also suppress developmental defects in *Polr1D* mutant larvae. To test this, we fed wt and *Polr1D* mutant larvae all three concentrations of NAC or vehicle control at 36 h AEL ( **Figure 1A, right** ). This timepoint was chosen because it is the earliest that we can reliably identify *Polr1D* mutant larvae (see Materials & Methods). At 96 h AEL, most *Polr1D* mutant larvae treated with vehicle were stalled in the L1 larval stage, whereas wt larvae progressed to the L3 larval stage as expected ( **Figure B ix-xvi** ). Treatment of *Polr1D* mutant larvae with NAC did not suppress larval developmental arrest or growth defects and had no effect on wt larvae ( **Figure 1G, H, I** ). We speculate that the failure of NAC treatment to rescue the defects of *Polr1D* mutant larvae, unlike treatment of zebrafish embryos and early larvae with NAC (Ulhaq et al., 2024), could be due to irreparable DNA damage caused by elevated ROS levels during embryogenesis (prior to when we are able to treat larvae with NAC).

Here, we show that maternal antioxidant treatment reduces the severity of developmental and growth defects caused by an amino acid substitution in a clinically relevant residue of *Drosophila* Polr1D. This finding is consistent with the craniofacial rescue that maternal antioxidant treatment promoted in mouse models of TCS (Sakai et al., 2016). While mammalian systems remain essential for clinical relevance, they are resource-intensive and less suited for rapid chemical testing. Our *Drosophila* model offers a genetically accessible, fast, and low-cost *in vivo* system that can be used to facilitate the discovery and prioritization of candidate therapeutics for TCS.

## Methods

**Fly stocks and husbandry**

Flies were reared at 25°C and 60% humidity on a 12-hour light/dark cycle, on homemade food based on the Genesee Scientific Nutri-Fly MF formula: 25 g/L Inactive Dry Yeast (Genesee Scientific), 89.5 g/L Dry Molasses (Genesee Scientific), 57 g/L Fly Stuff Yellow Cornmeal (Genesee Scientific), 5.84 g/L Nutri-Fly Drosophila Agar (Genesee Scientific), and ∼0.064 M propionic acid (Sigma Aldrich).

To generate *Polr1D* mutant larvae, *Polr1D ^1^ /CyO, ActGFP* virgin females were crossed with *Polr1D ^Df^ /CyO, ActGFP* males, and *Polr1D* ^1^ / *Polr1D ^Df^* larvae (“ *Polr1D ^1^ /Df* ”) were identified by a lack of GFP expression at 36 h after egg-laying (AEL). Oregon-R-P2 (BDSC #2376) was the wild-type control for all experiments. All crosses and experiments were performed at 25ºC.

**Egg/larvae collections and Antioxidant treatment**

N-Acetyl- *L* -cysteine (NAC, Sigma-Aldrich PHR1098) was dissolved in 1X PBS to a concentration of 100 mM and diluted in 1X PBS to 1 mM and 0.01 mM. For larval treatments, NAC was added to active yeast to create a paste, and an equivalent amount of 1X PBS was added to active yeast as the vehicle control. For maternal treatments, NAC dissolved in 1X PBS had 1/20 ^th^ volume of active yeast thoroughly mixed in, before pipetting onto standard food and dried before use. NAC supplemented food was made fresh. Virgin females were placed on fresh NAC or vehicle control every 24 hrs, for 3 d prior to crossing.

Crosses were performed in cages with grape juice agar plates (Genesee Scientific) and yeast paste (without vehicle or NAC). Eggs were collected every 2 to 4 h, *Polr1D ^1^ /Df* larvae were selected at 36 h AEL, and 30-50 larvae were transferred to fresh grape juice agar plates with either NAC or vehicle yeast paste every 24 h. Larvae were collected at 96 h AEL for imaging and larval stage analysis.

**Larval analyses**

Larval area was calculated at 96 h AEL using images and the measure tools in Fiji, cohorts of 10 larvae were imaged for three biological replicates for a total of n=30 (Walker et al., 2025). Larval cuticles were transferred to microscopes slides with 50-100 µl of a solution of 3:1 lactic acid: sterile water, covered with a coverslip, and incubated overnight at 65°C. Coverslips were sealed and mouth hooks were imaged under DIC optics, larval stage was determined based on the morphology of the larval mouth hooks, using both mouth hook size and the number of teeth in each mouth hook (Palumbo et al., 2022).

## Reagents

**Table:** 

***Drosophila* Strains **	**Genotype**	**Derived from**	**Available from**
* Polr1D ^1^ /CyO, ActGFP *	w*; Polr1D ^1^ pr ^1^ /CyO, P{w ^+mC^ =ActGFP}JMR1	BDSC #3304 and BDSC #4533	Palumbo et al., 2022
* Polr1D ^Df^ /CyO, ActGFP *	w*; Df(2R)Exel8040/CyO, P{w ^+mC^ =ActGFP}JMR1	BDSC #7847 and BDSC #4533	Palumbo et al., 2022
Oregon-R-P2	Wild-type		BDSC #2376

**Table:** 

**Chemicals**	**Available from**
N-Acetyl- *L* -Cysteine (NAC)	Sigma-Aldrich PHR1098
